# Treatment‐related experiences and preferences of patients with lung cancer: a qualitative analysis

**DOI:** 10.1111/hex.12417

**Published:** 2015-10-15

**Authors:** Ines Aumann, Kristine Kreis, Kathrin Damm, Heiko Golpon, Tobias Welte, J. Matthias Graf von der Schulenburg

**Affiliations:** ^1^ Center for Health Economics Research Hannover (CHERH)Leibniz University of HannoverHannoverGermany; ^2^ German Center for Lung Research (DZL)HannoverGermany; ^3^ Department of Respiratory MedicineHannover Medical SchoolHannoverGermany

**Keywords:** chemotherapy, experiences, lung cancer, preferences, qualitative interviews, treatment

## Abstract

**Background:**

Lung cancer is one of the most common types of cancer worldwide, and it causes significant challenges for patients due to the poor survival rate and treatment‐related side‐effects. Because of lung cancer's great burden, identification and use of the patients' preferences can help to improve patients' quality of life.

**Objective:**

Interviews with patients who have lung cancer were used to ascertain a range of experiences and to make recommendations regarding the improvement of treatment based on these patients' preferences. Because chemotherapy is the common treatment option for lung cancer, we focused on this treatment. The interviews were audio‐taped, verbally transcribed and evaluated via content analysis.

**Setting and Participants:**

A total of 18 participants (11 men and 7 women) with small or non‐small‐cell lung cancer who were receiving chemotherapy in one clinic were interviewed between June and July 2013.

**Results:**

Two main aspects with different subthemes were identified during the interviews. One main aspect focused on organizational context, such as the treatment day process, or experiences with different stakeholders, such as with the health insurance company or physicians. The other category referred to experiences that influenced psychosocial factors, including physical and mental experiences.

**Discussion and Conclusion:**

Patients reported different experiences concerning physical, psychological and organizational areas during chemotherapy. Nevertheless, some potential areas for improving care, and therefore the quality of life of patients with lung cancer, could be identified. These improvement measures highlighted that with small, non‐time‐consuming and inexpensive changes, the treatment for patients with lung cancer can be improved.

## Introduction

Lung cancer is one of the most common cancers with 1.8 million cases worldwide in 2012, and it was the leading cause of cancer‐related death.^1^ In relation to other cancers, lung cancer has a poor 5‐year survival rate. According to the different severities at diagnosis, the rate is between 2% for patients with distant metastases, 16% for patients with cancer in the lung and nearby lymph nodes and 49% for local lung cancer.^2^

Different types of treatment for lung cancer exist. Depending on the severity of the disease, surgery, chemotherapy or radiotherapy are potential treatment options. Nevertheless, chemotherapy is often undertaken either alone or in addition to surgery. The treatment causes a high burden for patients with cancer, including physical complaints about the disease itself, side‐effects of the therapy, mental stress, and a lessening of family life and leisure activities. Other affected areas include professional limitations, financial worries, the need to apply for support services, the integration of inpatient and outpatient therapy treatment measures, and regular interaction with physicians and medical personnel.^3^

Corbin and Strauss pointed out that cancer diseases require a high degree of services from the private and medical setting of those affected.^4^, ^5^ Recent studies show that physicians often evaluate the needs and preferences of their patients in a different manner to the patients themselves.^6^, ^7^, ^8^ Although the ramifications of the patient's perspective on their disease are not a new discovery, it still has a strong presence in recent studies. Most studies focus on quantitative analyses and include the use of a standardized questionnaire. For example, Mühlbacher *et al*. 9 used a discrete choice experiment to ascertain patient preferences in relation to treatment of non‐small‐cell lung cancer (NSCLC). They pointed out that patients prefer an increase in ‘progression‐free survival’ and a reduction of ‘tumour‐associated symptoms’ (e.g. cough, pain). With these instruments, it is difficult to assess the broad range of experiences and preferences that people have had with their treatment or disease because only some attributes can be evaluated. Furthermore, a qualitative approach can give broader insight and a more in‐depth understanding of the experiences and preferences with chemotherapy. Qualitative studies of the burden and experiences of cancer patients with chemotherapy also exist but they mostly focus on cancer in general and only some studies integrated patients with lung cancer.^10^, ^11^, ^12^, ^13^

Due to the great burden of the disease itself and the effects of the treatment, it is necessary to assess patients' treatment‐related experiences to help optimize their quality of life. Therefore, this study focuses on the following two questions:

1. What is the particular burden for lung cancer patients with regard to treatment?
2. Which recommendations for improving treatment can be derived from the patients' preferences?

## Methods

We conducted semi‐structured, guideline‐based, face‐to‐face interviews with patients suffering from NSCLC or small‐cell lung cancer. The sample was recruited in cooperation with the oncology outpatient day clinic at the Hannover Medical School (MHH), Germany. The clinic covers the whole spectrum of medical treatments of a centre of supramaximal care with a total of 1518 beds and 452 783 patient contacts per year.

Patients were only included if they had undergone palliative chemotherapy at the time of the study and had experienced at least one cycle of chemotherapy. Patients who received adjuvant chemotherapy were excluded. The study nurse, who was not part of the treatment team, asked those patients to participate in the interviews. She provided information about the study's aim, the voluntary nature of consent, data collection and data processing. A confidential and anonymous handling of all personal data was promised. All information about the study and the declaration of consent was handed to the patients. To record different experiences, our maximum variation sample included patients from various social backgrounds, ages and treatment methods. Some patients were accompanied by relatives during the interview. Owing to financial and time‐related restrictions, the interviews were undertaken in the MHH's rooms. After written informed consent was obtained, most patients were interviewed in the time lag between the blood sample and chemotherapy in the oncology outpatient day clinic. All interviews were conducted between June and July 2013. A total of 18 patients with lung cancer were interviewed by a research assistant of the Center for Health Economics Research Hannover. The number of interviews was not predefined. We stopped conducting interviews after no new messages emerged. The study was approved by the MHH's Committee for Clinical Ethics.

The interviews were structured using a guideline based on information from the literature, which was developed in conjunction with an interdisciplinary group of researchers. Therefore, we conducted a systematic literature review from 12 electronic databases (e.g. EMBASE, MEDLINE), and included 20 qualitative and quantitative studies published between 2000 and 2012. This guideline was divided into four sections and contained open questions that encouraged patients to talk about their treatment‐related experiences and preferences in their own words. First, patients were encouraged to describe an average treatment day in hospital. Second, patients were asked to talk about their expectations and experiences concerning chemotherapy in general, as well as the most harmful and burdensome side‐effects. These questions targeted the experiences and effects of the chemotherapy on different areas, including physical or mental status, impact on daily living and contact with other patients. Third, patients were also encouraged to name ways of improving health‐care quality. The fourth section served as a way of potentially addressing an important topic for the patient. Additionally, some demographic data (e.g. age, or smoking and working status) were obtained before the interview. The interviews lasted 1–1.5 h and were audio‐taped. Each interview was completely verbally transcribed and anonymized. As interviews were conducted in German, the citations were translated by two professional translators who are native speakers. Disparities were clarified bilaterally.

Data were analysed using qualitative content analysis methods with the additional inclusion of inductive categories.^14^, ^15^ To ensure the accuracy of the analysis process, two researchers (Kreis and Aumann) read the interviews and paraphrased the relevant text independently using the MAXQDA program. A codebook was also generated. The researchers analysed the text on the basis of deductive categories, which were derived from the questions in the guide. The inductive categories were developed directly from the text. In addition, some sections of the interviews were discussed by an interdisciplinary research group to identify further inductive categories.

To obtain an overall impression of the content, the transcripts were read and re‐read. In subsequent discussions, the researcher checked the codes for consistency and agreement, and resolved any differences by an iterative process. The aim of a content analysis is to identify the cross‐relationships, repetitions, commonalities, and differences of the statements to demonstrate a trend regarding the results. To achieve this, all interpretations and arguments are documented and supported by citations.

## Results

### Participants

A total of 18 patients (11 men and 7 women) with lung cancer completed the interviews. The average ages were 75 years for the men and 63 years for the women. Three of the 18 patients had a small‐cell lung cancer diagnosis and 12 patients received additional radiation therapy. All patients were at a higher disease stage (>IIIA) due to their late diagnosis. Further demographics and cancer status are described in Table 1.

**Table 1:** Participant socio‐demographics and clinical setting

Number	Age, Gender	Diagnosis	Stages	Radiation	Chemotherapy
1	75m	non‐small	IV	yes	Second‐line therapy, 9 cycles; intravenous
2	68m	Small	IV	no	Second‐line therapy, 2 cycles; intravenous
3	61f	non‐small	IV	yes	Third‐line therapy, oral
4	63m	non‐small	IV	yes	Second‐line therapy, 2 cycles, intravenous
5	48f	non‐small	IV	yes	First‐line therapy, oral
6	74m	non‐small	IV	no	First‐line therapy, oral
7	59f	non‐small	IV	yes	First‐line therapy, 10 cycles; intravenous
8	70f	non‐small	IV	yes	First‐line therapy, oral
9	69m	non‐small	IV	no	Fourth‐line therapy, 2 cycles; intravenous
10	65m	Small	IV	no	First‐line therapy, 1 cycle; intravenous
11	76f	non‐small	IIIA	no	First‐line therapy, 2 cycles; intravenous
12	65m	non‐small	IV	yes	Second‐line therapy, 2 cycles; intravenous
13	60m	non‐small	IIIB	yes	Third‐line therapy, 2 cycles; intravenous
14	72m	non‐small	IV	yes	Third‐line therapy, 7 cycles; intravenous
15	61f	non‐small	IV	yes	Fifth‐line therapy, oral
16	70m	non‐small	IV	yes	Second‐line therapy, 97 cycles; intravenous, maintenance therapy
17	75m	small	IIIB	yes	Second‐line therapy, 2 cycles; intravenous
18	67f	non‐small	IV	no	Second‐line therapy, oral

f = female, m = male.

### Themes

During the content analysis of the interviews, we identified four main themes. The first theme describes the chemotherapy‐related experiences and preferences of patients with lung cancer in relation to the organizational aspects of their treatment, especially the day they receive chemotherapy. The second and third themes focus on experiences with different stakeholders (physicians and the health insurance company) and the last category contains treatment‐related experiences and preferences that influence psychosocial factors, including physical and mental experiences, and changes in the patient's social environment.

#### Theme 1: Experiences and preferences during the treatment day

All patients described a very similar course of treatment, which is characterized by the collection of a blood sample, a consultation with the physician to discuss the blood values and to determine the next treatment steps, the collection of the chemotherapy substance at the pharmacy, and the subsequent chemotherapy. This treatment procedure was described by patients in a clear and factual language without many breaks. For some patients, it is an ordinary day of treatment due to the large number of therapy cycles and it is described as a natural process. Regarding the treatment day, some patients also reported long waiting times, particularly between individual treatment steps.

[…] and then, normally, a blood sample is first taken, two ampoules, and it is sent to the laboratory, then comes an appointment with the physician, in which the lab results are discussed once again, and he decides whether or not chemo will be performed, you understand? And yes, then comes another waiting period. One has to come down here and register, then wait, then the pharmacy delivers the chemo mixture and that can take a while and, oh well. (P9, 69m)

Nevertheless, the burden of waiting times was perceived differently among the patients. Those who had a long distance to travel to the hospital perceived the waiting time as a large burden because the driver had to allow time for the treatment and journey. This also limited the patients' flexibility and freedom, and ensured that the patients were dependent on others. Most patients mentioned the waiting times but accepted them and considered them to be unimportant, irrelevant, or a small problem compared to other problems. Thus, they approved of the waiting times and thought it impossible to accelerate individual treatment sections.

Yes, but what the heck, because I think that [the waiting period] is all stuff that takes a back seat. In that respect, they can't possibly please everybody. (P4, 63m)

However, the patients did make suggestions for design improvements regarding the waiting times. First, patients wished for greater privacy during chemotherapy, such as through the provision of more treatment rooms, smaller rooms, or the inclusion of extra curtains. Second, patients requested more rooms so that no patient has to wait in the corridor before starting chemotherapy. Third, some patients wanted beds, or more rooms or comfortable chairs, while another patient would welcome the provision of headphones and music during chemotherapy. All these suggestions indicate that patients want to feel comfortable and need privacy.

Perhaps they should […] hand out earphones for music or something like that, don't you think? Then it wouldn't be so monotonous, one would get drowsy at the same time and doze a little, but with a little music in your ears, that's not a bad idea, is it? (P15, 61f)

#### Theme 2: Experiences with physicians:

As patients spend much time waiting for chemotherapy, meaning a highly stressful situation, they wanted to feel comfortable.

I do not know […], but you are nervous and you also have fears, isn′t it so? The clinic should be a place where you get the help to ignore the medical stuff and to relax. (P15, 61f)

Therefore, the organizational conditions, such as those mentioned above, and the personal relationship with the staff members, especially the physicians, must be suitable. Overall, the interviewed patients were very satisfied with the physicians. They trusted their physician regarding treatment decisions and felt unable to request improvements regarding the therapies.

[…] the doctor already has to know how to improve that [the therapy]. (P11, 76f)

However, during the interviews, some patients expressed thoughts on improvements regarding the contact and communication with physicians. Patients wished for a certain level of continuity with the physicians and a frequent change of physicians was criticized because the patients had to build up confidence again.

What I still regret is that one has just built up a rapport with a physician and he disappears overnight. (P2, 68m)

Chemotherapy is a tense situation. Routine and trust in physicians can help the patients to cope better with this situation. Patients often only knew the name of the senior physician, because other physicians frequently changed. Therefore, the confidence base of the patients that was given referred exclusively to the senior physicians. Patients know about the difficulties for a hospital to structure a treatment plan so that everybody always has the same contact person, but at least they wanted to be informed by the senior physician or another known staff member about any changes in the treatment responsibility. Nevertheless, the frequency of physician changes should be kept to a minimum.

For patients who have regular contact with one physician, conversations are important to build up trust and reduce fears. Some patients mentioned that the physician took substantial time with them and created a very personal atmosphere during conversations.

Well, as far as the physicians are concerned, they have a lot to do, they are really overworked, aren't they? But I must say that I am impressed with the physicians here. They have really taken their time and put in a lot of effort. Recently, I had an appointment with Dr. A, shortly before he left the clinic. He devoted more than an hour to me. Which physician allows more than one hour for a patient? (P3, 61f)

This personal atmosphere is an important prerequisite when it comes to the provision of information and its content. As patients are often overwhelmed by the range of information available from the internet, friends, and family, they need the help of a health‐care professional like a physician to select correct and important information.

It was shown that patients generally feel well‐informed by the physician about the treatment. Some patients, however, wanted more information about the handling and treatment of side‐effects to acquire more security in dealing with disease specific situations. In particular, they were interested in whether the doctor was the right contact for the different side‐effects and what therapies are available to combat them.

With regard to the dermatological history, one should really know from the very beginning, who to turn to if eczema or something else really appears. (P18, 67f)

Likewise, regarding types of communication, patients had different desires. One patient did not wish to receive information via telephone. This patient feared a dispensation of personal contact and the possibility to talk about potential problems face‐to‐face. However, other patients preferred shorter methods of communication, because they had already experienced a long journey from their homes to the hospital and the side‐effects of the chemotherapy cause high physical strain.

I called today and said: Yes, the radiotherapy comes to an end tomorrow. What happens now? Should I have another CT and where? Here or there? What do I need to take with me? I was told: ‘Yes, on Thursday – the day after tomorrow – here at the clinic. Then we can discuss it’. But that came from the office, not from the physician. Now, I ask myself, is that absolutely necessary. Because there are no facts available, absolutely nothing. If I came here and some tests had been done or a CT had meanwhile delivered a result, and they had wanted to discuss that with me face to face, then okay. I don't really think one wants to do that on the phone. But only so that you will probably be told: ‘Yes, see to it that you get a referral for a CT from your GP and get the necessary blood tests for the CT done. And as soon as you have the results, come and see me again’. That would have been more logical in my opinion. (P1, 75m)

Altogether, this section shows that, besides organizational aspects, physicians also play an important role in giving patients a trustful atmosphere during chemotherapy and to make them feel comfortable. Therefore, they need a continuous contact person who is informed about the disease and treatment. Furthermore, new physicians should be introduced to the patients by the contact persons. The physician could improve the confidence by taking enough time for treatment discussions, asking patients how they want to get information, and to concentrate more on the patients′ individual needs and personality. This could create enough transparency to increase the acceptance of the organizational structure, such as waiting times, and reduce the patients' fears.

#### Theme 3: Experiences with health insurance

In terms of the organizational context, patients often had experiences with other stakeholders, especially regarding health insurance. For patients with lung cancer in particular, the absorption of travel costs was of great importance. As patients are not allowed to drive or they feel unable to drive to the therapy themselves, they need a taxi or a relative to drive them. Due to most patients being unable to work, paying for a taxi is an additional financial burden. Because of this, patients can apply for reimbursement of taxi rides to the chemotherapy sessions using their health insurance. The interviews showed that most communication and settlement between the health insurance company and patients is simple and straightforward. In many cases, patients were supported by the applications made by hospital employees or physicians. Nevertheless, sometimes there were coordination problems with the health insurance companies, which were perceived as particularly troublesome by the patients. One patient reported that taxi rides for computer tomography (CT) were not approved as they were not part of the chemotherapy, and the patient would not ‘beg for a benefit’.

The taxi fares to chemo were covered, but those to CT, for example, were not, so I had to ask my girlfriend if she would drive me because I can't afford a taxi. Who can afford a taxi there and back? Hey? And if we have to come to the clinic twice a week, without receiving chemo or radiotherapy, who pays for that? (P3, 61f)

Equally, another patient was not compensated for the rides because of a treatment option available in another hospital, which was closer to the patient's home but not a certified centre. Another patient reported that the health insurance company had verbally confirmed they would finance the services, but subsequently refused until a new request was made. One patient reported problems completing the applications because she did not know whether the disease was chronic. As a result, she accidentally made false statements, consequently had to file an objection, and incurred considerable expenses. In addition, the long waiting time for the granting of support services through health insurance was criticized. Altogether, the patients who have had bad experiences with health insurance feel overwhelmed with the administrative burden because they never had in such an extend contact to the health insurance before, and so this situation is new for them. Without help from nurses and physicians, the situation for the patients would further deteriorate and, therefore, they wish to have support from the health insurance.

#### Theme 4: Treatment‐related experiences and preferences of the patients that influence psychosocial factors

Besides the experiences with the organizational factors and stakeholders, the chemotherapy had an influence on the patients' psychosocial situation. The patients reported many physical side‐effects, such as general sickness, low load capacity, and absence of appetite due to the chemotherapy. Problems with changes in their external appearance because of hair loss or skin rashes were also mentioned.

These side‐effects caused great physical limitations resulting in lower performance levels and flexibility. As a result, patients reported a decrease in sporting and household activities. Additionally, patients often were unable to continue with their work. This situation occurred very suddenly and, thus, changed the patients' daily routines. Combined with their inability to work, some patients were worried about their financial security and economic existence, especially self‐employed patients. Some of them even had to apply for early retirement due to their illness.

Well, let me say this: I have been written off work and I suddenly have to spend the whole day at home. I have been ripped out of my environment, my professional life. (P5, 48f)

Whether someone can afford it is an issue that relates to the economic situation, or if it is someone who is on the dole who gets cancer. That is actually another (unclear) aspect of this illness, that one is drained financially. So, if it causes us to lose our company now, which we feared at the beginning, that would be a disaster. (P4, 63m)

The changes in their daily life along with the fears resulting from the disease and the treatment cause psychological effects.

Yes, I'm at the end of my strength. I can hardly move, can hardly walk, can hardly breathe. If I didn't have my girlfriend, I wouldn't be able to do anything, hey? (P3, 61f)

The psychological effects are characterized by different feelings. Patients differ between hopes and fears. On the one hand, the patients wish that the chemotherapy helped and extended their life but, on the other hand, they are afraid of physical disabilities, a lack of flexibility, and loss of independence. Due to these psychological strains, patients develop various strategies to deal with these limitations.

One group of patients took every opportunity to go for a walk and undertook specific breathing exercises. These patients wanted to actively take part in life and keep in touch with family and friends.

I try to increase right now my walking distance so that I go out and walk around (P7, 59f)

Another group of patients stayed at home and cut themselves off from their external environment. These patients often reported changes in mood and that they sometimes behaved defensively and aggressively.

It isn′t interesting anymore. I watch no news. It is all the same to me whatever happens anywhere in the world (P4, 63m)

Despite these differences between the two groups of patients, family is an important factor for both. They need support from the family to deal with the disease but they do not want to be a burden to their family. Nevertheless, patients report positively about the family growing closer together and building a better relationship, although the family was shocked about the diagnosis and it is difficult for them to deal with the situation.

The family recognized if somebody does not feel well, then you must stick together. (P5, 48f)

There were, however, quite contradictory experiences concerning the circle of friends. Some patients distanced themselves from their friends and in some cases lied to them in order to avoid talking about their real problems.

Well, the behaviour of friends that you spend time with is of course always a little/they said it again today, everyone always says ‘Oh, you're looking good!’ And then I think to myself: ‘Oh! I don't want to hear that word again!’ Because it's always such a poor little cancer patient, as though all of them walk around with bald heads or wigs, which constantly remind everyone of the situation. So one is/I am never free of the situation in that sense. (P18, 67f)

However, for patients without a family, their circle of friends was of great importance, providing household support or rides to hospital. For these patients, friends were indispensable. Altogether, chemotherapy leads to high physical and psychological strain for the patients. Strategies for dealing with these problems differ between the patient groups. Nevertheless, contact and support from family plays an important role for patients.

## Discussion

Patients with lung cancer have had a variety of experiences that have affected their physical, psychological, and organizational areas of life. During the interviews, the patients with lung cancer sometimes directly reported their preferences to support and improve treatment. In addition, based on the patient‐reported experiences, further recommendations can be derived. This section focuses on improvements to the treatment of patients with lung cancer, and distinguishes between patients' reported wishes and recommendations based on their reported experiences, in which some criteria mutually influenced each other. In other words, an improvement in organizational factors could, for example, enhance mental factors too.

First, some patients complained about long waiting times during chemotherapy and desired a more acceptable design of waiting times. This included greater privacy, such as through extra curtains or smaller treatment rooms. Other studies also identified the waiting time as an important aspect,^10^, ^16^, ^17^ because patients get frustrated, angry, and irritated. Mitchell *et al*.'s results show that patients think that the ‘delay in the clinic might be caused by adverse events, staff shortages and the general pressure of the throughput of patients’.^10^ Conversely, in our study, patients expressed the opinion that the waiting time could not be reduced but, instead, better shaped. Nevertheless, it could be an option to reduce the waiting time during chemotherapy by the family doctor taking a blood sample 1 day earlier so that the patients start the treatment day by directly discussing the treatment plan with the physician in the clinic. As the waiting time would be reduced, this could also improve the situation for the accompanying person.

Second, some patients reported problems communicating with their health insurance company concerning travel costs. Therefore the health insurance should optimize their quality and time of advice for these patient groups. Another option is to use and integrated these problems into the existing structure in the clinics. Although patients have the support of the physicians and nurses, this is not always sufficient. The capacity for so‐called case managers, which are often located at the clinic, should be increased so that they have more time to go through the application documents together with the patients. However, a systematic review of the use of such measures to optimize cancer care pathways shows that case management is a black box, and it is not clear which areas contribute to an improvement of the pathways, due to different or unclear definitions.^18^ Therefore, the case manager could have a gatekeeper function to optimize the treatment's structure, or the function of an advocate to answer labour and social law questions. It is also possible for the health insurance company to provide additional consultancy services that are specialized in treatment‐related problems for patients with cancer using health insurance.

Third, the interviews showed the patients' general satisfaction with their physicians. Leydon *et al*. 19 confirmed this relationship of trust by patients with cancer. Frequently changing physicians in the clinic was perceived negatively by patients, and a German study reached the same conclusion.^20^ To improve the patients' understanding of this situation, physicians should look for an open and honest conversation with patients and should respect their personality.

Fourth, patients with lung cancer reported different preferences regarding forms of communication. Some patients preferred personal contact with the physicians, while others favoured communication via telephone. In particular, those patients with a long distance to travel wanted to receive information via telephone. Thus, to communicate with patients in their preferred way, physicians should ask their patients at the beginning of the therapy which method of communication they want to use.

In addition, patients required more information about the treatment of side‐effects. Comprehensive information about the chemotherapy itself existed, but there was a lack of clear treatment options for possible side‐effects. Clinic staff should advise who the appropriate contact partners are for the patients. A further possibility would be to integrate patients into an interdisciplinary ‘tumour board’. The National Cancer Institute defined a tumour board as a ‘treatment planning approach in which a number of doctors who are experts in different specialties (disciplines) review and discuss the medical condition and treatment options of a patient.^21^ This includes medical, surgical and radiation oncologists. Within such a meeting, patients could be truthfully informed about side‐effects and treatment options by specialists. Better treatment of side‐effects could also positively influence patients' abilities to participate in work and social life. This tumour board should be convened during the process of therapy decisions, as well as in the course of individual treatment steps, as patient‐reported experiences might be relevant for the subsequent treatment steps.

Fifth, another fear that patients had was the financial burden, not only because of the inability to work, but also due to the indirect costs, for example those caused by searching for a driver to the clinic. This form of stress was associated with a high psychological burden for patients and their family. Timmons *et al*. 22 also confirm these results in a qualitative analysis of patients with breast, lung and prostate cancer. Thus, greater support in the household and subsidies for taxi expense could lessen this burden.

## Limitations

Some study limitations need to be acknowledged. This study was conducted in one large clinic with a centre of supramaximal care, which limits the transferability of the organizational findings to other, especially smaller, clinics. Nevertheless, the organizational process of chemotherapy is largely standardized, particularly in centres with certification, which means that the organizational aspects may not be different in other clinics. Furthermore, some patients had prior experiences with other clinics. As this study only included patients from the German health‐care system, the transferability of the experiences with the health insurances is limited. Additionally, the interviews were conducted in the rooms of the oncology outpatient day clinic. Patients may have answered questions incompletely or dishonestly. However, as the interviewer was not a member of the clinic, she may have been more likely to create an atmosphere of trust compared to a clinic member. Finally, a selection bias may have affected the results because we could not interview patients whose state of health did not allow study participation. This group of patients could have had other treatment‐related experiences and different preferences for chemotherapy.

## Conclusion

This study analysed the burden for patients with lung cancer caused by the treatment. Compared to other studies, we identified relevant experiences that influenced the atmosphere and well‐being of patients with lung cancer during chemotherapy. Therefore, we identified that the experiences with organizational processes, health insurance, physicians, and physical and psychological side‐effects influenced the patients' preferences. Furthermore, we used the identified experiences and preferences to derive recommendations about how the treatment can be modified. Based on their experiences, the following potential areas for improvement were defined: changing the waiting times, providing more information about the side‐effects of the treatment options, making individual arrangements regarding communication methods between the physician and patient and improving information about the changing physicians during treatment. With these changes, patients could feel better during chemotherapy and have fewer fears so that their quality of life could be improved. They are also more likely to accept organizational limitations, such as waiting times.

## Conflicts of interest

No conflicts of interest have been declared.

## Source of funding

Federal Ministry of Education and Research (BMBF); Heinemannstraße 2, 53175 Bonn.
